# Structural optimization and hydraulic performance analysis of bionic pit flow channels based on a genetic algorithm

**DOI:** 10.1038/s41598-022-26569-1

**Published:** 2022-12-17

**Authors:** Tianyu Xu, Yanru Su, Zhouming Su, Shuteng Zhi, Ennan Zheng

**Affiliations:** School of Hydraulic and Electric Power, Heilongjiang University, Harbin, 150080 China

**Keywords:** Fluid dynamics, Mechanical engineering

## Abstract

Orthogonal experiments have mostly been used in the structural optimization of drip irrigation emitter flow channels. To further improve the efficiency of the optimal design, this study used a genetic algorithm to optimize the structure of the bionic pit flow channel. Based on the structural similarity and performance optimization of the torus-margo bordered pit structure, the constitutive equation of the flow channel unit was constructed. The selection, crossover and mutation operators were set by the genetic algorithm, and the objective function value was calculated. The design variables and known variables that met the requirements were put into the computational domain model, and the pit flow channel structure was simulated and optimized. The results showed that there were large low-velocity regions at the junctions and corners of the pit flow channel units at a working pressure of 50 kPa, and no complete low-velocity vortices were observed, indicating that the flow channels had good anti-clogging performance. The distribution of flow velocity on the same cross-section was quite different, which made the flow layers collide and mix, which intensified the loss of energy, indicating that it had a good energy dissipation effect. The multivariate linear regression analysis showed that the four variables of tooth stagger value (*j*), flow channel angle (*θ*), tooth spacing (*l*) and inner and outer boundary spacing (*h*) had a decreasing degree of influence on the flow index (*x*). The flow index (*x*) was negatively correlated with the tooth stagger value (*j*), flow channel angle (*θ*) and tooth spacing (*l*), and positively correlated with the inner and outer boundary spacing (*h*). The test results of physical samples showed that the average error between the simulation results and the real values was 3.4%, indicating that the accuracy was high, which can provide a basis for the structural optimization design of related pit drip irrigation emitters.

## Introduction

As a core component of drip irrigation systems, the structural parameters of flow channels play an essential role in uniformity and anti-clogging^1^. The optimization of the labyrinth flow channel aims to reduce the flow index, enhance the energy dissipation and improve the anti-clogging ability^2^. The flow index reflects the sensitivity of the flow regime and flow rate to pressure changes, which is directly affected by structural parameters and is an important factor reflecting its hydraulic performance^3,4^. The flow index of drip irrigation emitters is typically between 0.5 and 0.6, and the hydraulic performance still has much room for improvement^5^. Many scholars have proposed new design ideas for structure optimization and developed new drip irrigation emitters with different flow channel structures^6,7^. Zhang et al.^8^ formulated a multiobjective optimization problem for the trapezoidal labyrinth flow channel structure, and solved the optimization problem with the same shape and different sizes. Xing et al.^9^ proposed a perforated drip irrigation emitter (PDIE) based on the structure of scalariform perforation plates in plant xylem vessels. The energy dissipation mechanism was further verified by numerical simulation and physical experiments, and it was noted that the PDIE had the best hydraulic performance in the high-pressure zone. Jin et al.^10^ constructed a new double gear rectangular labyrinth channel by adding internal teeth. The hydraulic performance of the two emitters was analysed through numerical simulation. The results showed that the hydraulic performance of the double gear rectangular labyrinth channel drip irrigation emitter was better.

The data analysis of drip irrigation emitter structure optimization is mostly based on orthogonal experiments. Li et al.^11^ studied the relationship between the hydraulic performance and structural parameters of bidirectional flow channel drip irrigation emitters through orthogonal experimental design. Chu et al.^7^ used numerical simulation methods combined with orthogonal experiments to study the influence of structural parameters on hydraulic performance. Yuan et al.^12^ combined the orthogonal experimental method with grey target theory to carry out multiobjective optimization of the flow channel. The preferred values obtained by conducting orthogonal experiments can only be some combination of the levels used in the experiments, and the preferred results will not exceed the range of the levels taken. In addition, they cannot provide a clear direction for further experiments, so orthogonal experiments are not a perfect solution.

In this paper, a bionic design of a pit flow channel drip irrigation emitter was designed and studied^13^. The flow index was analysed based on the swarm intelligence optimization algorithm^14^, a type of genetic algorithm, and the corresponding structural parameters were calculated and verified by numerical simulation and experimental testing. The specific objective of the present study is to (1) examine the influence of the structural parameters of pit drip irrigation emitters on the flow index, (2) evaluate the flow velocity and pressure distribution of the flow channel, and (3) establish a predictive model for the flow index. The results provide a reference for the optimal design of flow channel structural parameters.

## Research methods and principles

### Emitter flow channel unit structure design

#### Sample selection

Conifers are some of the largest biological communities at present. The flow inside the xylem depends on the adjacent tracheids for water transport. The junction of tracheids is a low-resistance water transmission channel constructed by the pit structure. Conifers are gymnosperms, and the junction is a torus-margo bordered pit structure^15–17^.

**Figure 1:**
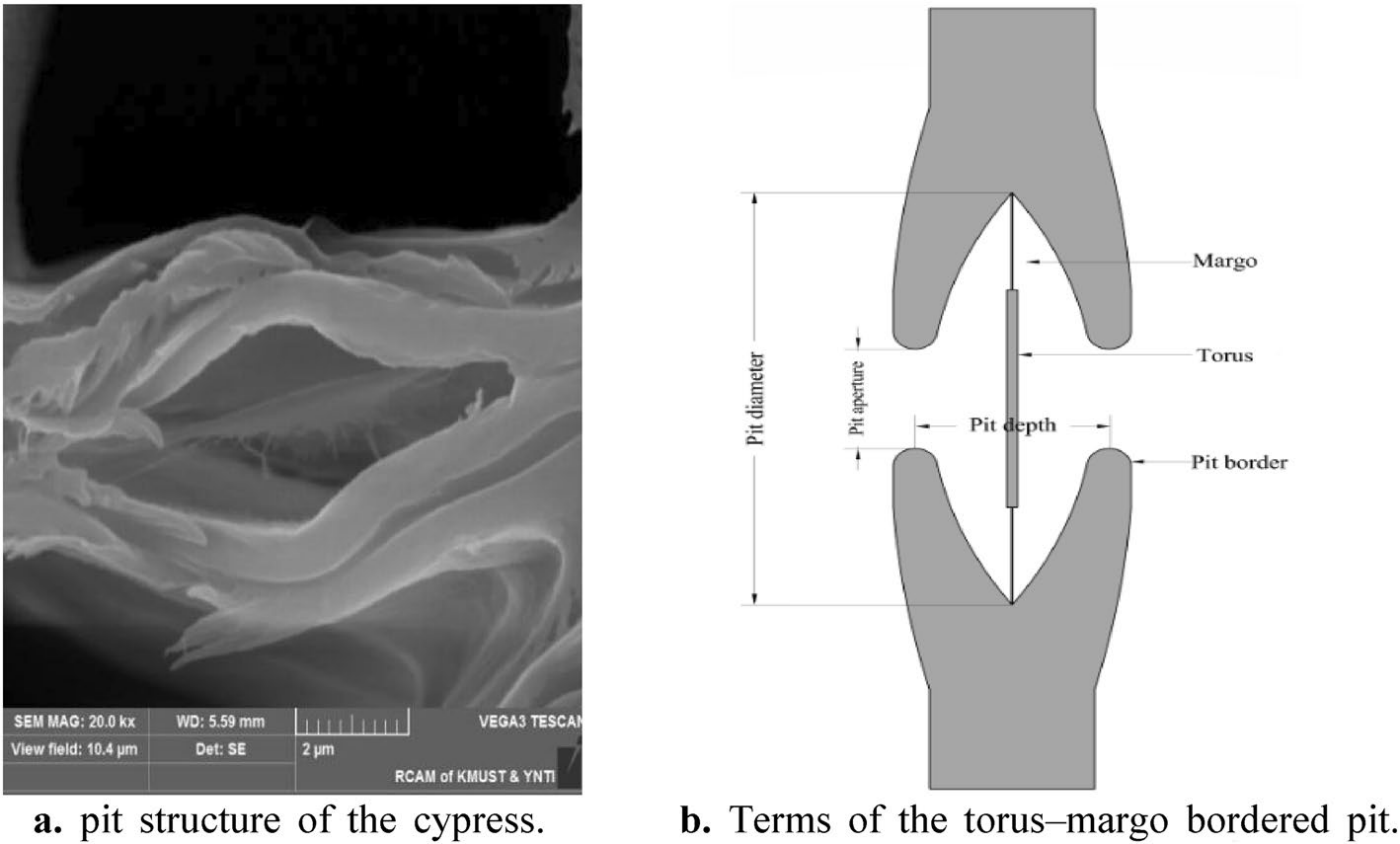
(**a**) pit structure of the cypress. (**b**) Terms of the torus–margo bordered pit.

The geometry of torus–margo bordered pit structures in the xylem tracheids of cypress trees was obtained by scanning electron microscopy (SEM) (Fig. 1a). The various characteristic terms of the torus–margo bordered pit structure are shown in Fig. 1b. The torus–margo bordered pit structure in plant xylem tracheids has good pressure drop stability. Therefore, the torus–margo bordered pit structure of cypress was selected for bionic optimization design.

#### Flow channel unit structural parameters

**Figure 2:**
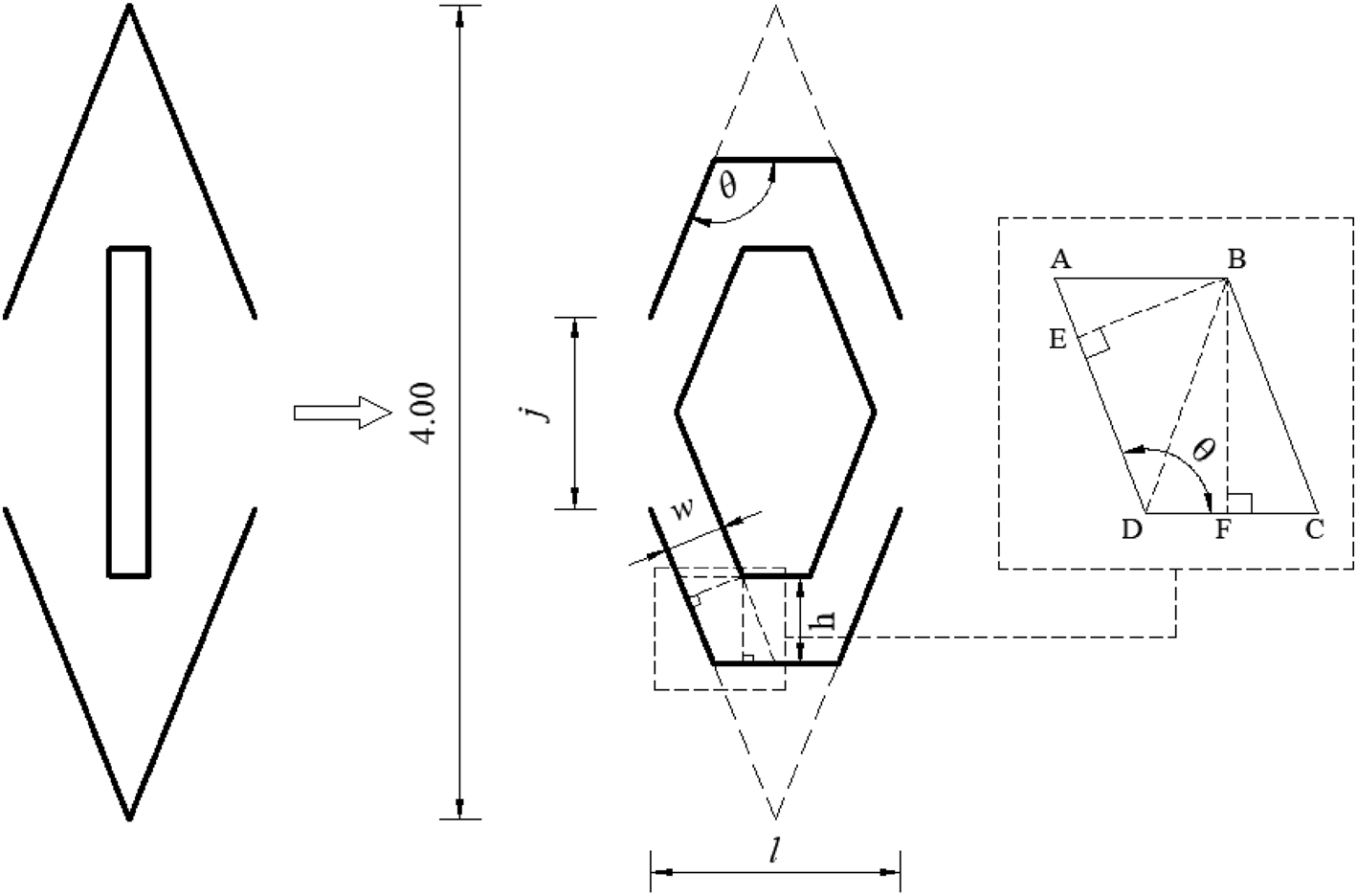
Bionic design of pit flow channel structure. (*l*) is tooth spacing, (*h*) is inner and outer boundary spacing, (*θ* ) is the flow channel angle, (*j*) is tooth stagger value, (*w*) is the width of the flow channel, A, B, C, D, E, and F are the letters marked in the derivation of the equation and have no practical significance.

Combined with the features of the torus–margo bordered pit structures in Fig. 1, the pit flow channel drip irrigation emitter was designed based on structural similarity and performance optimization^13^. Figure 2 shows the bionic design of the pit flow channel structure. The flow channel structural parameters are shown in Fig. 2, where (*l*) is the tooth spacing, (*h*) is the inner and outer boundary spacing, (*θ*) is the flow channel angle, (*j*) is the tooth stagger value, and (*w*) is the flow channel width. (*l*), (*h*), (*θ*) and (*j*) are the basic parameters of the flow channel structure^18^. The number of flow channel units (*N*) is fixed at 12, and the flow channel depth (*D*) is set to 0.6 mm. The distance between the intersection points of the flow channel outer boundary extension lines is fixed at 4 mm. When the intersection point of the flow channel inner boundary extension lines is located at the midpoint of the outer boundary, the flow channel width (*w*) can be determined as shown in Eq. (1).

1 $$ w = \sqrt {\left( {\frac{l}{4} + h\cot \theta } \right)^{2} + h^{2} } \times \sin \left( {\theta - \arctan \frac{16h - 4jh}{{4l - jl - 4lh}}} \right) $$

According to the experiments and related literature^19,20^, the value ranges of (*l*), (*h*), (*θ*) and (*j*) in the Eq. (1) are shown in Table 1.

**Table 1:** The value ranges of structural parameters.

Parameter	Value
Tooth spacing *l*/mm	1.2–2
Inner and outer boundary spacing *h*/mm	0.4–0.75
Flow channel angle *θ*/(°)	120–150
Tooth stagger value *j*/mm	0–1

#### Relationship between pressure and flow rate of the pit flow channel drip irrigation emitter

The relationship between pressure and flow rate can be a good reflection of the hydraulic performance of the emitters, which is expressed by flow index^5^. The smaller the flow index of the drip irrigation emitter is, the better the hydraulic performance. In a certain pressure range, the relationship between flow rate and pressure can be expressed by Eq. (2).

2 $$ q = kH^{x} $$

where *q* is the flow rate at a certain pressure (L/h), *H* is the inlet pressure (kPa), *k* is the flow coefficient and *x* is the flow index.

#### Energy dissipation efficiency of the pit flow channel drip irrigation emitter

**Figure 3:**
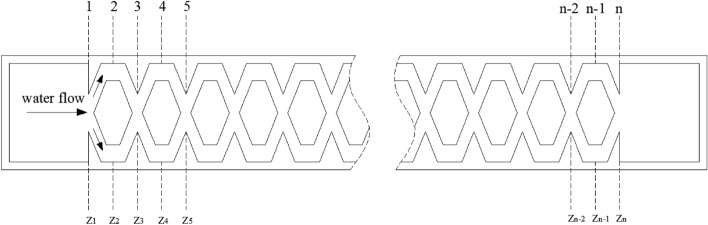
Schematic of water flow in pit flow channel.

The purpose of energy dissipation is to make the pressurized water eliminate the excess energy through the pit drip irrigation emitter, which can make the irrigation more uniform. There is no energy exchange between the inside of the emitter and the external environment in this study, and only friction head loss and local head loss occur in the flow. It can form diversions, sudden turnings, convergences and collisions to increase energy dissipation in the pit drip irrigation emitter. Combined with the relevant literature^21^, the energy dissipation efficiency of the emitters can be expressed by the energy dissipation coefficient. A schematic cross-sectional view shows the distribution of the pit flow channel in Fig. 3, where the arrow represents the direction of water movement.

The model used to analyse the energy loss in the pit flow channel was the Bernoulli equation. Assuming that the flow between numbered sections satisfied the Bernoulli equation, the energy conservation between the adjacent two sections can be expressed in Eq. (3).

3 $$ \begin{gathered}   \frac{{P_{1} }}{{\rho g}} + \frac{{V_{1}^{2} }}{{2g}} + z_{1}  = \frac{{P_{2} }}{{\rho g}} + \frac{{V_{2}^{2} }}{{2g}} + z_{2}  + \xi _{1} \frac{{V_{2}^{2} }}{{2g}} + \lambda \frac{{l_{1} V_{2}^{2} }}{{2Dg}} \hfill \\   {\text{ }}\frac{{P_{2} }}{{\rho g}} + \frac{{V_{2}^{2} }}{{2g}} + z_{2}  = \frac{{P_{3} }}{{\rho g}} + \frac{{V_{3}^{2} }}{{2g}} + z_{3}  + \xi _{2} \frac{{V_{3}^{2} }}{{2g}} + \lambda \frac{{l_{2} V_{3}^{2} }}{{2Dg}} \hfill \\   \;\;\;\;\;\;\;\;\;\;\;\;\;\;\;\;\;\;\;\;\;\;\;\;\;\;\;\;\;\; \cdots  \cdots  \hfill \\   \;\;\;\;\;\;\;\;\;\;\;\;\;\;\;\;\;\;\;\;\;\;\;\;\;\;\;\;\;\; \cdots  \cdots  \hfill \\   \frac{{P_{{n - 1}} }}{{\rho g}} + \frac{{V_{{n - 1}}^{2} }}{{2g}} + z_{{n - 1}}  = \frac{{P_{n} }}{{\rho g}} + \frac{{V_{n}^{2} }}{{2g}} + z_{n}  + \xi _{{n - 1}} \frac{{V_{n}^{2} }}{{2g}} + \lambda \frac{{l_{{n - 1}} V_{n}^{2} }}{{2Dg}} \hfill \\  \end{gathered} $$

where *P*_*n*_ and *V*_*n*_ are the average pressure and flow velocity at section *n*, *ρ* is the fluid mass density, *g* is the acceleration of gravity, *z*_*n*_ is the position head of the water flow at the section, *ξ*_*n-1*_ is the local loss coefficient from section *n*-1 to section *n*, *λ* is the friction factor of head loss, *l*_*n-1*_ is the length between two adjacent sections and *D* is the hydraulic radius of the rectangular section flow channel and is defined as

4 $$ D = \frac{A}{\chi } = \frac{my}{{2\left( {m + y} \right)}} $$

In which *m* and *y* are the width and depth of the flow channel section. Adding the two sides of Eq. (3) gives the following formulation:

5 $$ \frac{{P_{1} - P_{n} }}{\rho g} = z_{n} - z_{1} + \xi_{1} \frac{{V_{2}^{2} }}{2g} + \xi_{2} \frac{{V_{3}^{2} }}{2g} + \cdots + \xi_{n - 1} \frac{{V_{n}^{2} }}{2g} + \lambda \frac{{LV_{n}^{2} }}{2Dg} $$

where *l*_1_ + *l*_2_ + *l*_3_ + … + *l*_*n*-1_ = *L*, is the total length of the pit flow channel. The positioning head is zero due to the horizontal flow path with *z*_1_ = *z*_2_ = *z*_3_ = … = *z*_*n*_. The continuity equation of the flow is stated as

6 $$ V_{1} A_{1} = V_{2} A_{2} = V_{3} A_{3} = \cdots = V_{n} A_{n} $$

where *A*_*i*_ (*i* = 1,2,…,*n*) is the flow sectional area of the corresponding section. A substitution of Eq. (6) into Eq. (5) obtains:

7 $$ \frac{\Delta P}{{\rho g}} = \left[ {\lambda \left( {\frac{{A_{1} }}{{A_{n} }}} \right)^{2} \frac{L}{D} + \sum\limits_{i = 1}^{n - 1} {\xi_{i} \left( {\frac{{A_{1} }}{{A_{i + 1} }}} \right)^{2} } } \right]\frac{{V_{1}^{2} }}{2g} $$

Thus, a comprehensive energy dissipation coefficient of the flow channel is defined as

8 $$ \xi = \left[ {\lambda \left( {\frac{{A_{1} }}{{A_{n} }}} \right)^{2} \frac{L}{D} + \sum\limits_{i = 1}^{n - 1} {\xi_{i} \left( {\frac{{A_{1} }}{{A_{i + 1} }}} \right)^{2} } } \right] $$

Consequently, Eq. (7) is simplified as

9 $$ \frac{\Delta P}{{\rho g}} = \xi \frac{{V_{1}^{2} }}{2g} $$

Further, the comprehensive loss coefficient is expressed as

10a $$ \xi = \frac{2}{{V_{1}^{2} }} \cdot \frac{\Delta P}{\rho } $$

Or written as

10b $$ \xi = \frac{{2m^{2} y^{2} }}{{q^{2} }} \cdot \frac{\Delta P}{\rho } $$

where *ξ* is the energy dissipation coefficient, *m* is the width of the flow channel structure (m), *y* is the depth of the flow channel structure (m), *q* is the average flow rate of the flow channel cross-section (m^3^/s), *Δp* is the pressure at the inlet (Pa) and *ρ* is the density of water (kg/m^3^).

### Genetic algorithm and optimization principle

Optimization of flow structure parameters should obtain the optimal value of multiple parameters within a certain range. To solve the optimization problem of multiple structural parameters in the flow channel quickly, the genetic algorithm in the gradient-free optimization method is used to design the correlation optimization^14,22^. The genetic algorithm (GA) is a computational model of the biological evolutionary process that simulates the mechanism of natural selection and genetics of Darwinian biological evolution^23,24^, and it is a method to search for the optimal solution by simulating the natural evolutionary process. A genetic algorithm is a type of global optimization algorithm^25^. Selection, crossover and mutation operators cooperate and compete with each other so that the objective function value can be converted to obtain information about the adaptive value.

Joint optimization of the flow channel structure by the genetic algorithm and computational fluid dynamics was carried out as follows:

1. The constitutive equation between the structural parameters was constructed by the structural similarity and performance optimization of the torus-margo bordered pit structure.
2. The value ranges of the structural parameters were determined by referring to relevant literature^19,20^.
3. The selection, crossover and mutation operators were set in the genetic algorithm. The objective function values were calculated, and the design variables were generated.
4. It was determined whether the convergence condition was satisfied. If so, step 5 was executed. Otherwise, the algorithm returned to step 3, and the next generation of design variables was generated.
5. The design variables and known variables were input into the computational domain model to perform the simulation calculation.
6. The simulation results were output to fit the *q* ~ *H* equation and find the optimal solution, and the calculation was finished.

### Principle of multiple linear regression analysis and correlation tests

Multiple linear regression analysis is a statistical analysis method used to study the linear relationship between a dependent variable and multiple independent variables. The linear relationship between the flow index (*x*) and tooth spacing (*l*), inner and outer boundary spacing (*h*), flow channel angle (*θ*) and tooth stagger value (*j*) was established in this paper. Multicollinearity tests and significance tests were performed. The influence degree of each structural parameter on the flow index was obtained by observing the standardized regression coefficients.

The multicollinearity test is measured by the variance inflation factor (VIF), which represents the ratio of the variance of the regression coefficient estimator compared to the variance assuming a nonlinear relationship between the independent variables^26^. When VIF < 5, there is very little collinearity. When 5 < VIF < 10, the collinearity can be regarded as weak. When VIF > 10, the collinearity can be regarded as strong, and it is necessary to carry out certain treatments. The variance inflation factor (VIF) is defined as shown in Eq. (11).

11 $$ VIF_{i} = \frac{1}{{1 - R_{i}^{2} }} $$

where *R*_*i*_^*2*^ represents the R-square of the linear regression model of the i-th independent variable *X*_*i*_ with the other independent variables.

The F-test is used to detect whether the coefficients of the overall equation are significant. If the P-value of the F-test is less than 0.05, then the overall regression is significant. The regression equation^27^ is obtained from the unstandardized coefficients, and its form is shown in Eq. (12).

12 $$ y = \beta_{0} + \beta_{1} x_{1} + \beta_{2} x_{2} + \cdots + \beta_{m} x_{m} $$

where *y* represents the dependent variable, *x*_*1*_, *x*_*2*_, ……, *x*_*m*_ represent the independent variables, *β*_*0*_, *β*_*1*_, *β*_*2*_, ……, *β*_*m*_ represent the unstandardized coefficients and *m* represents the number of variables.

### Numerical simulation and boundary conditions

**Figure 4:**
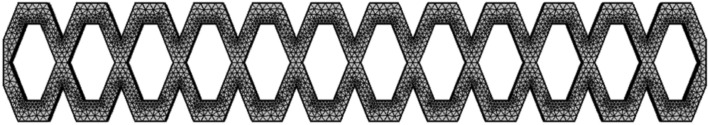
The grid division of emitter flow channel structure.

COMSOL software was used to simulate the pit flow channel, and the *k-ε* turbulence model of RANS was used in the fluid domain. The inlet pressure was set to 50 kPa and evenly increased to 250 kPa in steps of 25 kPa. The outlet was set as the outflow boundary, and the no slip condition was applied to the wall surface. The fluid domain was divided by unstructured meshes of tetrahedra and hexahedra, and a certain mesh refinement was performed at the corners of the flow channel structure^28^. According to the grid independence test based on the prediction accuracy of inlet and outlet pressure drop, the predicted pressure drop difference was 0.36% (as shown in Table 2), and it was assumed that the number of grids had no effect on the calculation results. The grid size was 0.0593–0.193 mm, and the total number of grids in the fluid domain was approximately 65,000. The pit flow channel grid is shown in Fig. 4.

**Table 2:** Grid independence test.

	Number of grids	Pressure drop value/(Pa)	Pressure drop difference
Very coarse	16,664	49,327	–
Coarse	33,623	49,640	0.63%
Standard	64,820	49,820	0.36%
Refined	106,235	49,965	0.29%

### Test method

**Figure 5:**
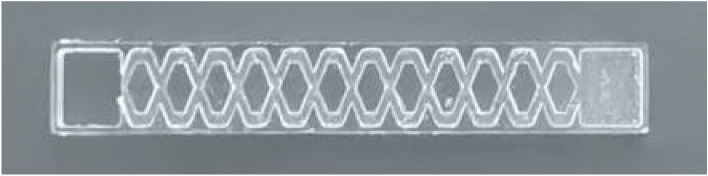
Physical drawing of the pit flow channel drip irrigation emitter.

In this study, Solidworks software was used to design the test model, and an EM-G32S-X32 high-precision engraving machine with a manufacturing accuracy of 0.01 mm and a repeatability accuracy of 0.005 mm was used to produce an equal-scale pit channel drip irrigation emitter. The model was made of Plexiglass. Three drip irrigation pipes were set up in the test setup, and each pipe was set up with three drip irrigation emitters, for a total of nine pit flow channel drip irrigation emitters^29^. During the test, the pipeline pressure was read by a 0.1 level precision pressure gauge. The pressure fluctuation range was less than 2%. First, the flow rate of the drip irrigation emitter was tested at a working pressure of 50 kPa, and then it was tested every 25 kPa at 1.0–5.0 times the working pressure. Each test time was 5 min, and each test was repeated 3 times. The water output was recorded with a 0.25 level precision measuring cylinder, and the average value was calculated. The relevant regression analysis was carried out, and the relationship curve between the flow rate and the pressure was obtained. The physical drawing of the pit flow channel drip irrigation emitter is shown in Fig. 5.

## Results

### Optimization results and simulation comparison

The genetic algorithm parameters were selected as follows: the initial number population size was 50; the selection operator used the roulette wheel; the crossover operator used the two-point crossover operator, and the probability was set to 0.75; the mutation operator used Gaussian mutation, and the probability was set to 0.02; the maximum number of genetic generations was 50. The optimization results of the structural parameters are shown in Table 3.

**Table 3:** Optimization results of the structural parameters.

Parameter	Value
Tooth spacing *l*/mm	1.22
Inner and outer boundary spacing *h*/mm	0.43
Flow channel angle *θ*/(°)	112.30
Tooth stagger value *j*/mm	0.94

**Figure 6:**
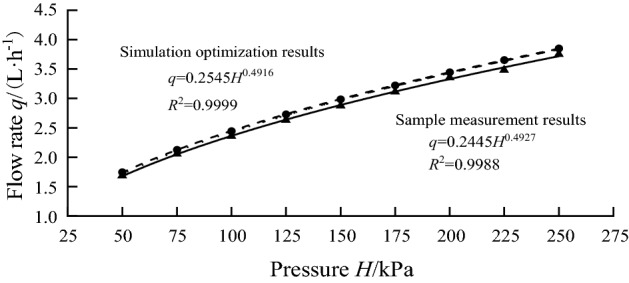
Comparison of flow-pressure curve between sample measurement results and simulation optimization results.

Using the optimal results, the simulation calculation was performed, and Eq. (2) was used for regression analysis to obtain the relationship between the flow rate and pressure. That is, *q* = 0.2545*H*^0.4916^, and the correlation coefficient was *R*^2^ = 0.9999, indicating that the model fit was good. Meanwhile, the energy dissipation coefficients of the emitters were calculated by Eq. (10b), and the results were in the range of 135.35–139.12, indicating that the model dissipated energy well. The relationship between the measured flow rate of the physical sample and pressure was *q* = 0.2445*H*^0.4927^, and the correlation coefficient was *R*^2^ = 0.9988. The flow-pressure curve comparison between the sample measurement results and the simulation optimization results is shown in Fig. 6. The measured flow rate was less than the simulated flow rate, which might be caused by the inaccuracy of the measurement method and losses in the model itself. However, the average error was 3.4%, which satisfied the relevant design requirements^30,31^. The correlation coefficient was 0.9995, and the P-value was much less than 0.01, indicating that the correlation was significant.

### Flow velocity distribution

**Figure 7:**
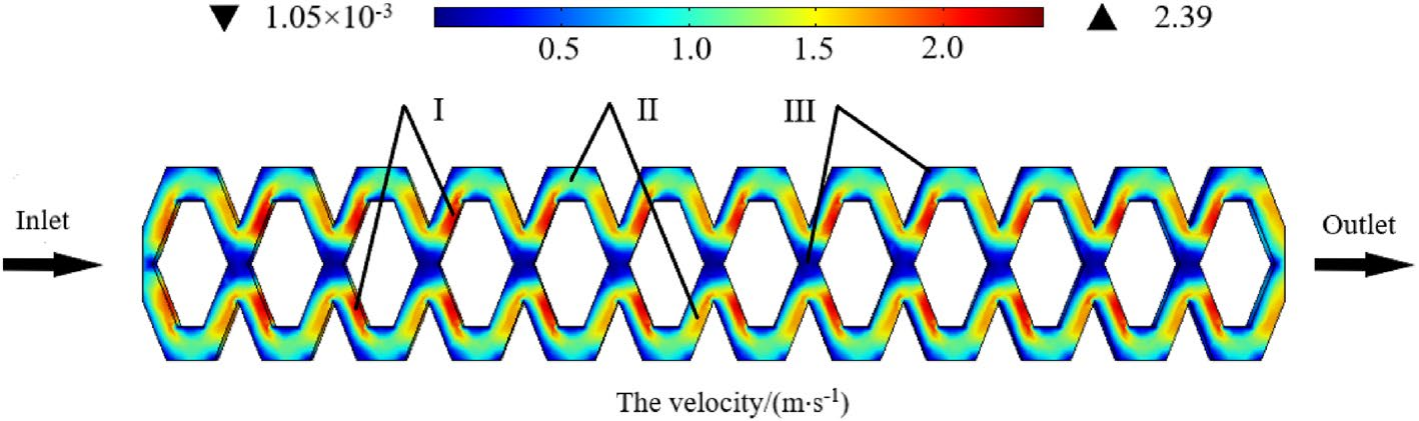
The velocity distribution in the pit flow channel.

The COMSOL postprocessing clearly showed the flow velocity distribution of each position at 50 kPa operating pressure. The different velocities in Fig. 7 are shown in different colours, from blue to red, indicating a gradual increase in velocity. The fluid velocities at all points in the flow channel were not the absolute velocity of the pit drip irrigation emitter. However, the relative velocities at different locations in the flow channel were valid. The minimum flow velocity was 1.05 × 10^–3^ m/s, and the maximum flow velocity reached 2.39 m/s. The flow velocity was roughly divided into three parts: Area I was the high flow velocity area, where the velocity was in the range 1.75–2.39 m/s; Area II was the medium flow velocity area, where the velocity was in the range 0.75–1.75 m/s; Area III was the low flow velocity area, where the velocity was below 0.75 m/s. Figure 7 shows that the flow velocity distribution of each flow channel unit was similar. Much energy was consumed from Area I to Area II and Area III, indicating that the energy dissipation effect was strong.

**Figure 8:**
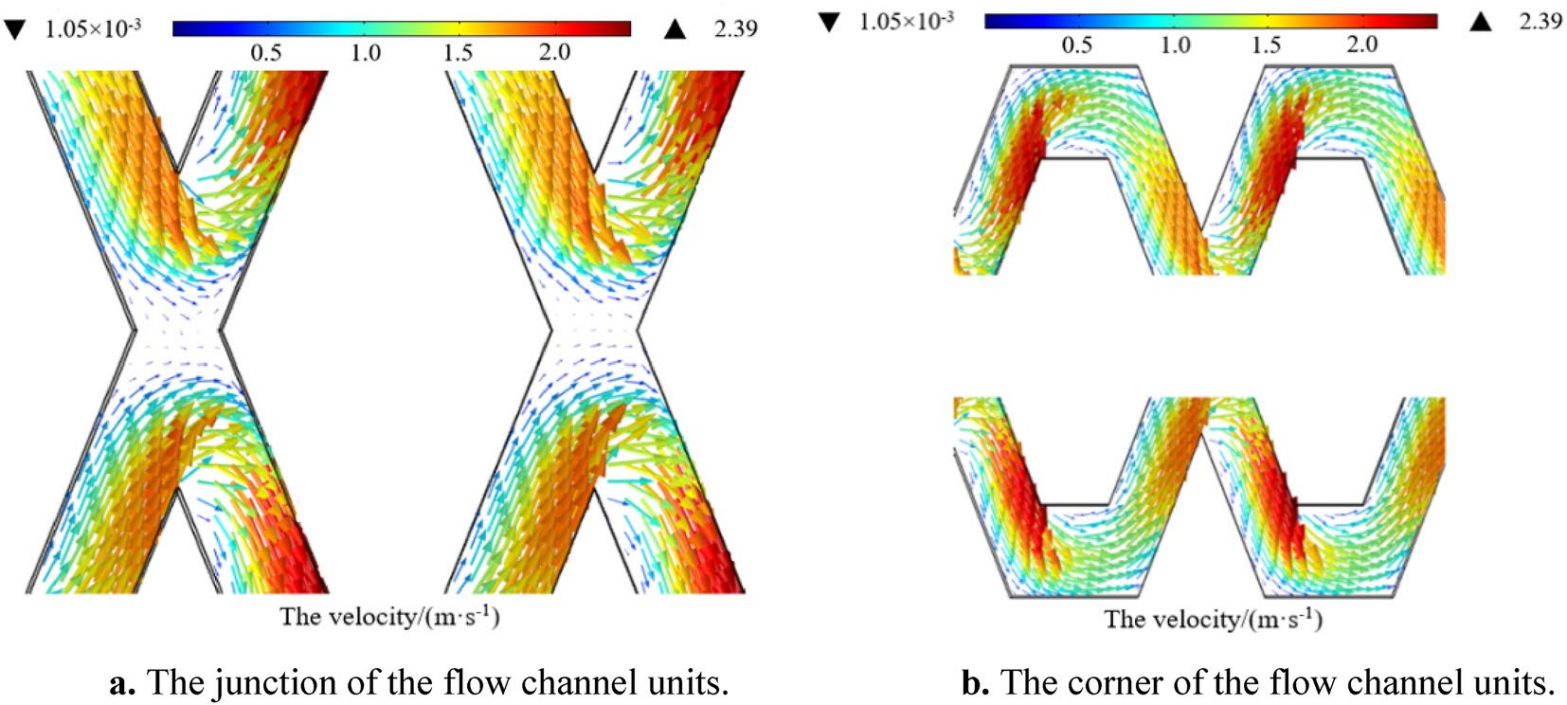
(**a**) The junction of the flow channel units. (**b**) The corner of the flow channel units.

Figure 8a,b show the flow velocity distribution at the junction and corner of the flow channel units, respectively, in which different velocities are represented by different colours. As shown in Fig. 8a, two streams of high-speed water collided at the junction of the flow channel units and rubbed against the surrounding walls, causing significant energy loss and very good energy dissipation. A low-velocity zone was generated at the junction centre of the flow channel units, which was very small and stable. When the flow was merged, it was divided into two new streams and entered the next channel unit.

As shown in Fig. 8b, the upper and lower structures in each flow channel were exactly symmetrical, so the cross-sectional area and flow velocity of the same section were equal. The corners of each flow channel unit were also in a state of low flow velocity. The reason was that when the water flow entered the corners, the cross-sectional area suddenly increased. According to the continuity equation, the flow velocity decreases. In addition, the water flow turned sharply at the corner of the flow channel units and collided violently with the wall, which further consumed energy and stabilized the water flow.

Therefore, the pit flow channel had a strong energy dissipation capacity, and its energy dissipation effect was very good. In addition, no complete low-velocity vortex current was observed at the junction or the corner of the flow channel, which indicated that the solid particles did not easily accumulate and cause clogging, and the pit flow channel had good anti-clogging performance. These combined factors made the pit flow channel drip irrigation emitter produce a stable drip flow.

### Pressure distribution

**Figure 9:**
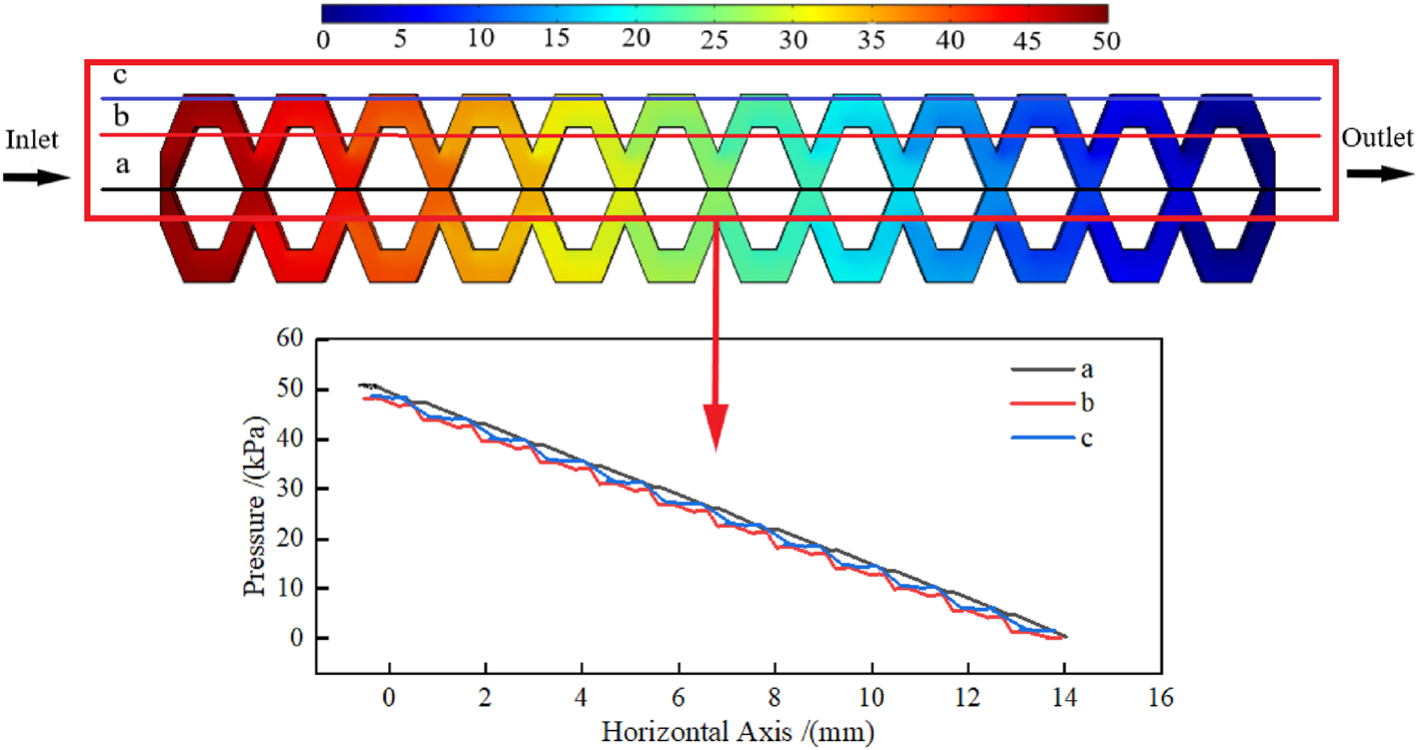
The pressure distribution in the pit flow channel.

The COMSOL postprocessing clearly showed the pressure distribution of each position at 50 kPa operating pressure. The different pressures in Fig. 9 are shown in different colours, from red to blue, indicating a gradual decrease from inlet to outlet. The fluid pressures at all points in the flow channel were not the absolute pressure of the pit drip irrigation emitter. However, the relative pressures at different locations in the flow channel were valid. The flow channel had only friction head loss and local head loss. There were twelve flow channel units in total, and each flow channel unit had the same shape and size. The pressure changed linearly along the direction of the flow channel, and the average pressure drop of each flow channel unit was approximately 4.17 kPa.

To understand the variation trend of the pressure drop at each position of the pit flow channel, three representative horizontal axes a, b, and c were taken on the same plane, and the pressure drop at the positions passed by the three horizontal axes was studied. Because the pit flow channel in this study had an up/down symmetrical structure, the three horizontal axes were all located in the upper half. The details are shown in Fig. 9. Figure 9 shows that the pressures on the three horizontal axes gradually decreased from the inlet to the outlet. When in the same horizontal position, the pressure on the three horizontal axes was a > c > b. Because the pressure is lower where the velocity is higher in a continuous fluid, and vice versa. Figure 7 and Fig. 9 show that horizontal axis a passed through the area with the lowest speed, and horizontal axis b passed through the area with the highest speed. In addition, horizontal axis c passed through the area with the middle speed. Therefore, the pressure on the three horizontal axes should be a > c > b at the same horizontal position. The horizontal axis a showed that the pressure drop was basically the same over the same distance in the process of pressure reduction, and the horizontal axes b and c also showed the same characteristic. These results indicated that the pressure drop of each flow channel unit was basically the same, and the water flow repeated with the same energy loss in each channel unit until it reached the outlet of the flow channel.

### The relationship between flow index and structural parameters

To analyse the influence of four design variables, tooth spacing (*l*), inner and outer boundary spacing (*h*), flow channel angle (*θ*) and tooth stagger value (*j*), on the flow index (*x*), linear regression analysis was performed by using SPSS software based on the data calculated by successive generations of genetic algorithms during the simulation and optimization process. The results of data processing are shown in Table 4.

**Table 4:** The results of the linear regression analysis.

Design variables	Unstandardized coefficient	Standardized coefficient	Tolerance	Variance inflation factor
Constant term	0.5974	–	–	–
Tooth spacing *l*/mm	− 0.0083	− 0.0392	0.4771	2.0958
Inner and outer boundary spacing *h*/mm	0.0152	0.0303	0.2523	3.9639
Flow channel angle *θ*/(°)	− 0.0004	− 0.0650	0.2968	3.3690
Tooth stagger value *j*/mm	− 0.0530	− 0.8798	0.7194	1.3900

According to Table 4, the mathematical model between the structural parameters and the flow index can be derived as shown in Eq. (13).

13 $$ x = 0.5974 - 0.0083l + 0.0152h - 0.0004\theta - 0.0530j $$

where *x* is the flow index, *l* is the tooth spacing (mm), *h* is the inner and outer boundary spacing (mm), *θ* is the flow channel angle (°) and *j* is the tooth stagger value (mm).

The determination coefficient of the pit flow channel model was *R*^2^ = 0.722, and the P-value was 0.004, which was less than 0.01, indicating that the model had a high degree of fit and a highly significant difference. This was shown to be statistically significant. The variance inflation factor (VIF) value of the inner and outer boundary spacing (*h*) was the largest. The value was 3.9639 and less than 5, indicating that there was no collinearity problem for the four variables in this model, and the model was relatively reliable. By comparing the standardized regression coefficients of the four design variables in Table 4, the degree of influence of the design variables on the flow index (*x*) from highest to lowest was obtained as follows: tooth stagger value (*j*), flow channel angle (*θ*), tooth spacing (*l*) and inner and outer boundary spacing (*h*). The standardized regression coefficient of the inner and outer boundary spacing (*h*) was positive, which indicated that increasing the inner and outer boundary spacing (*h*) can increase the flow index (*x*). The standardized regression coefficients of the three structural parameters, tooth stagger value (*j*), flow channel angle (*θ*) and tooth spacing (*l*), were negative. This indicated that decreasing the tooth stagger value (*j*), flow channel angle (*θ*) and tooth spacing (*l*) can increase the flow index (*x*).

## Discussion

There have been many ways to improve and enhance the hydraulic performance of emitters. Such methods include designing a new type of emitter, analysing the structure of the emitter, adjusting the inlet pressure, etc.^1,32,33^. Among them, the most important method is to optimize the flow channel structure of the emitters, which is the most direct and effective method to change the hydraulic performance^9,11–14,32,34^. In this study, based on the bionic design of the torus-margo bordered pit structure in the xylem tracheids of the cypress stem system, a pit flow channel drip irrigation emitter was proposed^13^. Four important structural parameters were selected, and the flow channel structure was optimized by a genetic algorithm and numerical simulation to explore the hydraulic performance of the pit drip irrigation emitter.

In this study, the sample measurement results were compared with the simulation optimization results, and correlation analysis was carried out. The correlation coefficient between the two results was 0.9995, and the average error was 3.4%. Xing et al.^9^ and Xu et al.^13^ also concluded that the measured flow rate of the sample was smaller than the simulated flow rate. One of the reasons may be that the measurement method was not accurate enough and there were some losses in the model itself. Another reason may be that the sample specimen cannot be machined to be absolutely smooth, which generated additional energy dissipation in the flow channel.

From the pressure distribution and velocity distribution, it can be seen that the pressure was relatively large and the velocity was relatively small at the junction of the flow channel units and the corners of the flow channel. This was caused by the collision and squeezing of the water flow at the junction of the flow channel units and the corners of the flow channel.

Under a working pressure of 50–250 kPa, the flow index of the pit flow channel drip irrigation emitter was 0.4916. However, under the same pressure conditions, the flow index of the labyrinth channel drip irrigation emitter was generally above 0.5^30,35^. The hydraulic performance of the pit flow channel drip irrigation emitter was better than that of the common labyrinth channel drip irrigation emitter in every pressure range. Thus, it can provide more stable and uniform water flow and effectively improve irrigation efficiency. Pit flow channel drip irrigation emitters have a similar energy dissipation mechanism as perforated drip irrigation emitters and two-way mixed flow drip irrigation emitters^9,34^. The pit flow channel drip irrigation emitters divided the flow into two streams in the same direction by blocking the torus structure. When two streams meet, violent mixing and collision occur, which consumes considerable energy. In addition, the water flow turned sharply and collided at the corners of the flow channel, which also increased the energy consumption.

In this paper, the primary and secondary order of the influence of various structural parameters on the flow index was obtained through linear regression analysis: tooth stagger value (*j*) > flow channel angle (*θ*) > tooth spacing (*l*) > inner and outer boundary spacing (*h*). Combined with the existing study, it was found that when exploring the influence of channel structure parameters on hydraulic performance, the channel depth mainly affected the change in the flow rate of the emitter and had little influence on the flow index^14^. However, consistent conclusions were not reached regarding other structural parameters that affected the flow index. The reason may be that the structural forms and the structural parameters of the flow channel were different. With the development of related research, the structural forms of emitters have become increasingly diversified, including triangular labyrinth channels^32^, perforated channels^9^, two-way mixed flow channels^34^, and trapezoidal labyrinth channels^29^, which all have different structural flow channel parameters.

## Conclusions

In this study, a genetic algorithm was proposed to optimize the structural parameters of pit drip irrigation emitters. Numerical simulation and model experiments were used to analyse the flow index and energy dissipation effect of the optimal structural parameters. Our conclusions are as follows.

1. The optimal structural parameters of the pit flow channel drip irrigation emitter were found by the genetic algorithm. The corresponding physical samples were made, and a reasonable experimental method was designed for testing and comparison. The comparison results showed that the correlation coefficient between the simulated results and measured results was 0.9995, and the average error was 3.4%. This indicated that the simulated results were highly accurate, which can provide a specific basis for the relevant structure optimization design.
2. Through the simulations and related calculations of COMSOL software, a large amount of data between the flow rate and flow index was obtained, which provided a guarantee for the establishment of related mathematical models. The regression model between the flow index and each structural parameter was fitted by using multiple linear regression analysis. The influence degree of each structural parameter on the flow index was clarified in descending order: tooth stagger value (*j*), flow channel angle (*θ*), tooth spacing (*l*) and inner and outer boundary spacing (*h*). The flow index (*x*) was negatively correlated with the tooth stagger value (*j*), flow channel angle (*θ*) and tooth spacing (*l*) and positively correlated with the inner and outer boundary spacing (*h*).
3. This paper studied the flow velocity distribution and pressure distribution at various locations inside the flow channel under working pressure (50 kPa). There were large areas of low flow velocity at the junction and the corner of the flow channel units, but no complete low-velocity vortex was observed, indicating that the flow channel had good anti-clogging performance. The flow velocity distribution on the same cross-section had a large gap, which made the liquid of different flow layers collide and mix with each other and intensified the energy loss. The pressure gradually decreased from the inlet to the outlet, indicating that the pit flow channel drip irrigation emitter had a good energy dissipation effect.

Due to the unique nature of the emitter structure of this study, the derivation conditions of the relationship equation between the flow channel structural parameters are more demanding and rigid. Therefore, the relevant conclusions drawn have certain limitations. To further improve the performance of pit drip irrigation emitters, the anti-clogging ability of particles with different sizes should be studied in the future.

## Data Availability

The data that support the finding of this study are available from the corresponding author upon reasonable request.
